# How Multifunctioning Joints Produce Highly Agile Limbs in Animals with Lessons for Robotics

**DOI:** 10.3390/biomimetics9090529

**Published:** 2024-09-03

**Authors:** Stuart C. Burgess

**Affiliations:** School of Electrical, Electronic and Mechanical Engineering, Bristol University, Bristol BS8 1TR, UK; s.c.burgess@bris.ac.uk

**Keywords:** multifunctioning, joints, limbs, muscle

## Abstract

This paper reviews how multifunctioning joints produce highly agile limbs in animals with lessons for robotics. One of the key reasons why animals are so fast and agile is that they have multifunctioning joints in their limbs. The multifunctioning joints lead to a high degree of compactness which then leads to a host of benefits such as low mass, low moment of inertia and low drag. This paper presents three case studies of multifunctioning joints—the human wrist joint, knee joint and foot joints—in order to identify how multifunctioning is achieved and what lessons can be learned for robotics. It also reviews the multifunctioning nature of muscle which plays an important role in joint actuation. A key finding is that multifunctioning is achieved through various means: multiple degrees of freedom, multifunctioning parts, over-actuation and reconfiguration. In addition, multifunctioning is achieved through highly sophisticated layouts with high levels of integration and fine-tuning. Muscle also makes an important contribution to animal agility by performing multiple functions including providing shape, protection and heat. The paper reviews progress in achieving multifunctioning in robot joints particularly for the wrist, knee and foot. Whilst there has been some progress in creating multifunctioning robotic joints, there is still a large gap between the performance of animal and robotic joints. There is an opportunity to improve the agility of robots by using multifunctioning to reduce the size and mass of robotic joints.

## 1. Introduction

This paper reviews how multifunctioning joints produce highly agile limbs in animals with lessons for robotics. Animals are well known to have superior performance to robots in the area of locomotion and hand skills [1,2]. One reason for the higher performance is superior control and sensing [1]. Another reason is that of multifunctioning materials [3]. This paper shows that multifunctioning skeletal joints are another key reason for the superior agility of animal limbs. The paper gives examples of how multifunctioning leads to high performance, identifies how multifunctioning is achieved and discusses what lessons can be learned for robotics.

Multifunctioning is widespread in nature. The majority of biological materials are remarkably multifunctional. For example, human skin has many functions including being stretchable, tough, self-renewing, self-healing, self-lubricating and insulating [4]. In addition, skin is host to blood vessels, nerves, sweat glands, hairs and multiple sensors [4]. There have been many studies on the multifunctioning nature of biological materials such as muscle [5], bone [6], nacre [7], feathers [8], plant cuticle [9], cactus spines [10] and synovial fluid [11]. 

Multifunctioning is also widespread in nature at the subsystem level. The mammalian mouth has multiple functions of breathing, eating, drinking and creating sound [12]. The mammalian penis has two major roles of urinating and mating [13]. Seals use their flippers for ground locomotion as well as swimming [14]. Some birds such as gannets use wings for powered flight in air as well as powered underwater swimming [15]. Water birds like ducks generally have feet that are optimised for both walking on ground as well as swimming. Animal blood circulation systems perform many functions including temperature control, clotting and transportation of multiple materials to multiple destinations [16]. Multifunctioning is also prevalent in microbiology such as proteins [17] and cells [18,19]. 

Multifunctioning is also common in animal joints. Most animal joints are multifunctioning with multiple degrees of freedom. For example, the mammalian vertebral column has important functions of providing flexibility in multiple degrees of freedom as well as carrying heavy loads and providing a protective structure for a complex network of nerves [20]. By being multifunctioning, the vertebral column is able to be remarkably compact and lightweight.

Multifunctionality is a very advantageous design feature because it reduces the number of subsystems and components and produces a compact design. Multifunctioning in joints leads to a high degree of compactness which then leads to a host of benefits such as low mass, low moment of inertia and low drag. It also leads to reduced energy demands and the ability to meet tight dimensional constraints. Multifunctioning joints also have the additional benefit that it often enables the animal or robot to perform multiple high-level functional tasks. There has been a growing desire to have multifunctioning capabilities in robots such as multifunctioning rescue robots [21], multifunctioning arms [22] and multifunctioning rehabilitation gloves [23].

Significant research effort has gone into producing multifunctional materials such as multifunctioning composites [24,25], multifunctioning coatings [26] and multifunctioning battery structures [27,28]. Some multifunctioning materials are especially suitable for robotics. For example, materials have been developed that transform two-dimensional laminate actuators into three-dimensional structural elements [29,30]. These materials can lead to greater compactness and functionality in robotics and other areas. However, there has not been so much work on bioinspired multifunctioning subsystems such as limb joints.

**Figure 1:**
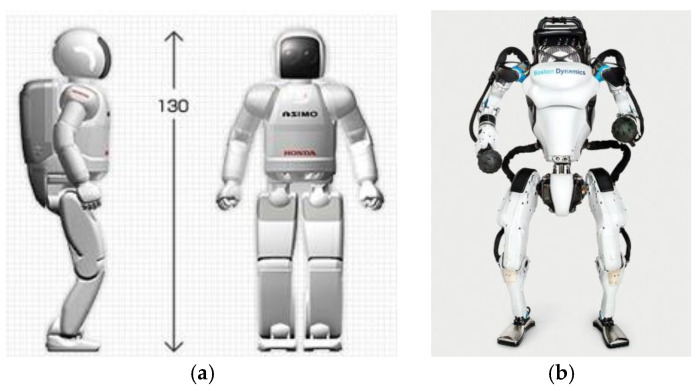
Two well-known humanoid robots. (**a**) Asimo [33]; (**b**) Atlas [34].

In comparison to nature, robotic joints tend to be bulky with less multifunctionality. For example, human prosthetic joints are relatively bulky with lower functionality when compared with the human joints they are replacing [31,32]. Two of the most well-developed humanoid robots have been the Asimo robot and the Atlas robot, as shown in Figure 1. These robots have some limbs that are reasonably compact, but they only achieve this due to reduced functionality in the joints. For example, the knee joints of the two robots cannot rotate around the tibia axis and the wrists cannot abduct–adduct. A key reason why robot joints are bulky and/or have less functionality is a lack of multifunctioning in the joints. 

Therefore, there is a gap in the research field in terms of the development of multifunctioning joints and there is potential benefit in learning from the multifunctioning strategies of biological joints. The review seeks to address this gap by analysing three key joints: the human wrist joint, human knee joint and human foot joints. These joints were chosen because they are highly multifunctioning and because they are of particular interest to the field of robotics. It also reviews the multifunctioning nature of muscle which plays an important role in joint actuation.

For each case study, the functions of the joint (or muscle) are defined and the means by which they are achieved is analysed and tabulated. Each section also reviews progress in achieving multifunctioning in robot joints. Key examples of multifunctioning bioinspired joints are given for the wrist, knee and foot. The paper makes recommendations for how to achieve greater multifunctionality in robot joints.

## 2. The Human Wrist Joint

### 2.1. Wrist Anatomy

**Figure 2:**
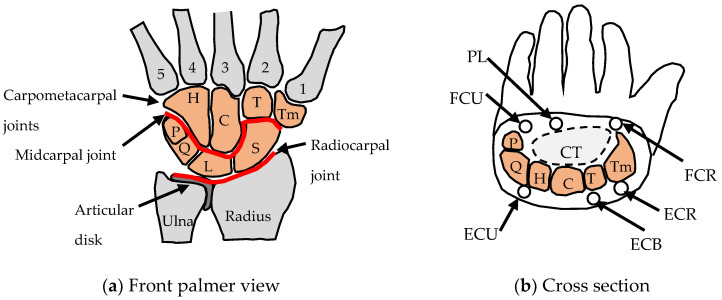
Schematic of the right wrist joint based on the anatomy in [35]. The eight wrist bones are highlighted. Key: H = hamate, C = capitate, T = trapezoid, Tm = trapezium, P = pisiform, Q = triquetrum, L = lunate, S = scaphoid. Main muscles: FCR = flexor carpi radialis, PL = palmaris longus, FCU = flexor carpi ulnaris, ECU = extensor carpi ulnaris, ECB = extensor carpi brevis, ECR = extensor carpi radialis, CT = carpal tunnel.

Figure 2 summarises the anatomy of the human wrist joint. The wrist has 8 bones, over 20 articulating surfaces [35] and over 25 ligaments [36]. The ligaments are stiff enough to provide stability under load but flexible enough to allow significant range of movement (ROM) [37]. The radius and ulna bones are connected by an articular disk (an oval plate of fibrocartilaginous meniscus) [38] (Figure 2a). There are six main muscle–tendon units that actuate the wrist (Figure 2b) [1] and these are located in the lower arm.

### 2.2. Requirements and Objectives

Table 1 summarises the four major functions of the wrist and the aspects of subsystem design that make multifunctioning possible. The design objective of the wrist is to form an assembly of parts that creates a biaxial (universal) joint at the same time as creating efficient load paths for in-plane and out-of-plane loads. An additional function of the wrist is to have proprioceptor sensors [39]. The wrist has been described as the most complex joint in the body due to the multifunctioning nature of the joint [40].

### 2.3. Wrist Functions and Common Parts

The wrist flexion–extension function is fulfilled with a double joint to produce a significant range of motion. The two joints are the radiocarpal joint and the midcarpal joint, as shown in Figure 2. The radiocarpal joint and midcarpal joints are ellipsoid in shape to enable abduction–adduction to occur as well as flexion–extension [35]. The third function of longitudinal load paths involves six of the wrist bones being aligned to create two compression load paths. The carpal arch has an important function of forming a protective passageway for the delicate tendons and nerves of the hand [42].

It is remarkable how the eight wrist bones perform four major functions. The six bones used in abduction–adduction are all used in flexion–extension. The capitate in particular is a key bone that plays a major role in all four functions. The axis of rotation for all wrist motion passes through the capitate. It is also particularly impressive that the carpal arch is perpendicular to the two arches in the plane of the hand.

### 2.4. Actuation and Fine-Tuning

The location of the four main wrist muscles (ECU, ECR, FCU and CRF) at the four corners of the wrist (Figure 2) means that they all can multifunction for both flexion–extension and abduction–adduction. For example, the ECU produces both extension and adduction and the FCU produces both flexion and adduction. The layout and curvature of the bones are such that there is an approximate common centre of rotation from the two joints for flexion and extension [43]. To produce a common centre of rotation, the radius of curvature at the radiocarpal joint has to be larger than the radius of curvature at the mid-carpal joint, with a unique proportion that gives a common centre of rotation. This results in a smooth reproducible joint motion. The geometry of the joint is also such that there is an approximate common centre of rotation for abduction–adduction [43]. The wrist bones are also well aligned with the metacarpals to produce a clear longitudinal load path from the metacarpals through the wrist bones to the lower arm with two main compression load columns [44]. This is beneficial because bone is stronger in compression than shear and tension [45].

### 2.5. Integration, Reconfiguration and Miniaturisation

The capitate is the most highly integrated part with at least five interfaces with adjacent bones, including a shear key (styloid process) where it interfaces with the third metacarpal bone in order to transfer shear loads. Reconfiguration occurs when the wrist joint is tensioned for increasing strength and stiffness under load. Examples of miniaturisation are found in sensors, nerves, blood vessels and synovial fluid. In the case of synovial fluid there is a special form of lubrication that creates a lubricant film between the sliding surfaces through an intricate combination of biomechanical and biomolecular factors acting at a range of scales [11]. The layout of wrist bones represents a unique solution where the layout performs multiple functions with multiple aspects of fine-tuning and integration. 

### 2.6. A Multifunctioning Robotic Wrist

**Figure 3:**
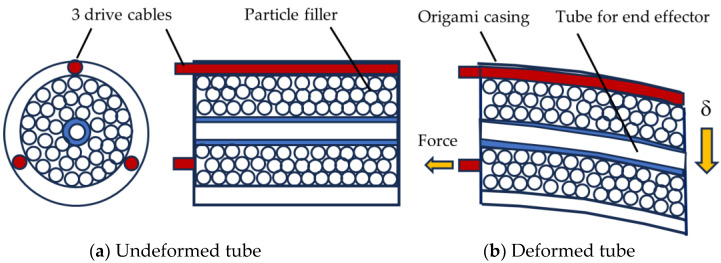
Bioinspired wrist concept using particles [46].

Robotic wrists are relatively bulky compared to the human wrist. In a review of 62 state-of-the-art prosthetic wrists, very few had the three degrees of freedom of the human wrist and those that did were all relatively very bulky [31]. One recent bioinspired wrist design that has improved compactness in the cross-section is showed conceptually in Figure 3 [46]. The design consists of a flexible tube of particles that can be deformed by three cables that surround the tube. The particles fill an annulus and create a stiffness that is low enough to be deformed but high enough to enable wrist functions. The tube can be made to bend in any direction by adjusting the relative tensions of the three cables. The cables are analogous to the tendons that surround the human wrist (Figure 2).

## 3. The Human Knee Joint

### 3.1. Knee Joint Anatomy

**Figure 4:**
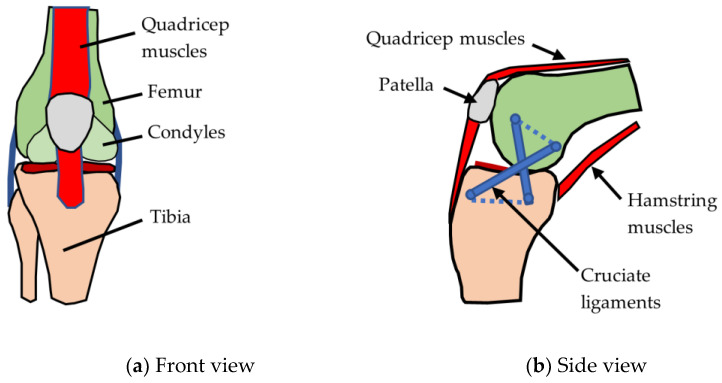
Anatomy of knee joint.

The main articulation in the knee occurs between the femur and tibia bones, as shown in Figure 4. The femur has two separate condyles with a gap in between to give space for the two cruciate ligaments that join the bones together and guide motion. There is also a sliding joint between the patella and a groove in the femur. The patella forms a low-friction joint that reacts the loads generated from the quadriceps muscle–tendon group. The patella also has a function of increasing the mechanical advantage of the quadricep muscles.

### 3.2. Requirements and Objectives

Table 2 summarises the four major functions of the knee joint and the aspects of sub-system design that make multifunctioning possible. The design objective of the knee is to create a joint with a large amount of flexion–extension movement but also with some movement in rotation [47]. The knee also has a function of locking in the fully extended position which helps reduce the effort involved in standing [48]. The knee must also be able to absorb shock loads during activities such as running and jumping. Other functions include load transfer for various external loads and position sensing [49].

### 3.3. Knee Functions and Common Parts

The flexion–extension function is carried out by the femur and tibia which form a cam-roller joint (Figure 3b). When the knee is in a position of flexion between around 30 and 90 degrees, the tibia is able to rotate axially (Table 2) relative to the femur with up to approximately 45 degrees external rotation and 25 degrees internal rotation [50]. Lesser rotations of the tibia can be achieved outside of the 30–90 flexion range. The tibia axial rotation function is important because it enables abilities like adjusting to uneven surfaces while running as well as general dexterity.

**Table 2:** Summary of knee functions and how they are achieved.

Main Functions	Flexion–Extension	Rotation	Locking at Full Extension	Shock Absorber
Schematic	Femur rolls against tibia 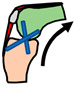	Geometry of joint allows rotation 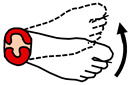	Geometry of joint allows locking 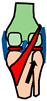	Meniscus absorbs shock 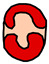
Common parts	Condyles Ligaments Meniscus	Condyles Ligaments Meniscus	Condyles Ligaments Meniscus	Condyles Meniscus
Actuation [51]	Seven muscles over-actuated	Five muscles over-actuated	One muscle for unlocking	
Fine tuning	Geometry of 4-bar linkage	Geometry of tibia plateau. Meniscus geometry	Joint geometry. Popliteus muscle alignment	Meniscus geometry
Integration	Ligaments, muscles and meniscus	Ligaments, muscles and meniscus	Ligaments, muscles and meniscus	Meniscus surrounding condyles
Reconfiguration			Unlocking with popliteus muscle	
Miniaturisation	Sensors, nerves, blood vessels, lubrication	Sensors, nerves, blood vessels, lubrication	Sensors, nerves, blood vessels, lubrication	Sensors, nerves, blood vessels, lubrication

The joint-locking function (sometimes called the screw home mechanism (SHM) [48]) locks the joint in full extension by maximising external rotation (outward rotation of the tibia) and tightening the cruciate ligaments. Locking is important because it gives stability and saves energy when standing. As the knee reaches full extension, external rotation is caused by the geometry of the condyles and by the lateral pull component of the quadricep muscles [47]. The shock absorber function is fulfilled by the meniscus. The meniscus is wedge-shaped on its inner edge and is sandwiched between the femur and tibia during load bearing. This significantly increases the contact area between the two bones, thus reducing contact stresses [52]. 

Like the wrist, the knee joint has a unique and complex geometry that enables it to perform several functions. The condyles and meniscus play a major role in all four functions. The meniscus performs multiple functions including acting as a dynamic guide for the femur, lubricating layer, nutrition provider and structure that withstands compression, tension and shear loads [52].

### 3.4. Actuation and Fine Tuning

The knee joint is over-actuated with more actuators than degrees of freedom. Over-actuation allows for fine-tuning of joint movements. In parallel with the cam-roller mechanism there is also a four-bar linkage mechanism [53], as shown in Figure 4b. The linkage mechanism is an inverted four-bar mechanism where the cross links are formed by the anterior and posterior cruciate ligaments (ACL and PCL). The parallel bars are effectively formed by the femur and tibia and these bars are represented schematically by dotted lines in Figure 4b. The four-bar linkage mechanism gives the knee a moving centre of rotation which enables the femur bone to roll around the tibia (Figure 4b) and hence produce large flexion angles [54]. The action of the four-bar mechanism also produces an advantageous increase in mechanical advantage during squatting [54]. 

The axial rotation function of the tibia is possible due to the geometrical clearance between the condyles on the femur bone and the tibial plateau and because the cruciate ligaments have lengths and orientations that allow a degree of rotation [50]. To maintain stability during flexion and extension, the meniscus translates together with the condyles of the femur bone. Translation of the meniscus is possible because the meniscus is anchored to the tibia by ligaments only on the outer edges of the crescent-shaped rings and not on the inner edges [52]. 

### 3.5. Integration, Reconfiguration and Miniaturisation

The parts of the knee joint are highly integrated, especially the meniscus in the way it surrounds the condyles. Joint locking and unlocking represents reconfiguration. To unlock the joint, the tibia is made to rotate internally by flexion muscles, especially the popliteus muscle behind the knee [47], as shown in Table 2. Like the wrist joint, there is miniaturisation in the sensors, nerves, blood vessels and lubrication system that helps to achieve compactness. The layout of the knee joint represents a unique solution where the layout performs multiple functions with multiple aspects of fine-tuning and integration.

### 3.6. A Multifunctioning Lockable Robotic Joint

**Figure 5:**
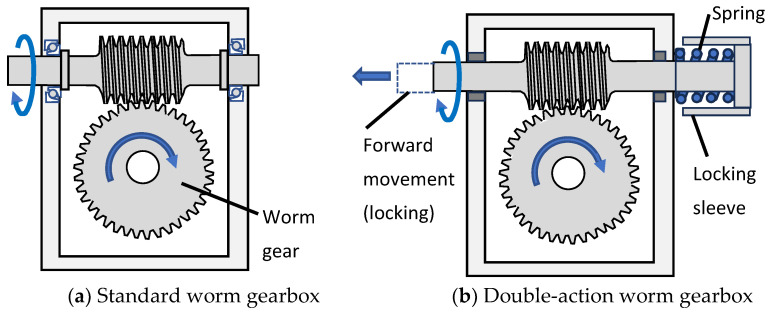
Multifunctioning double-action worm gearbox [55].

A multifunctioning lockable gearbox with analogies to the knee joint has been used on the robotic arm of ESA’s METOP satellite launched in 2019 [55,56]. Space satellites have extreme geometrical constraints due to the very limited space in rocket nose cones and so there is a strong desire to create compact designs through multifunctioning. The multifunctioning gearbox is shown in Figure 5b. A standard worm gearbox is shown in Figure 5a for comparison.

The double-action worm gearbox is able to perform three kinematic functions: (i) compliance during launch vibration like a rack and pinion gear; (ii) deployment of the appendage like a worm gear; and (iii) locking function at the end of deployment like a screw-nut. The locking sleeve is able to lock at the end of deployment because when the worm wheel can no longer rotate, the worm shaft screws forward until the locking sleeve comes into contact with the gear casing and engages with a high friction surface. The three gear functions are analogous to the three knee functions of tibia rotation, flexion–extension and locking, respectively. The locking function is analogous because like the femur, the worm wheel comes to the end of travel, and like the femur, there is a screw-home locking action.

The gearbox is called double-acting because the worm shaft has two degrees of freedom—rotation and translation. Interestingly, as with the knee joint, the spacecraft hinge is under-constrained, such that during deployment there are two degrees of freedom and the position of the hinge is a function of the spring reaction as well as the motor torque. This gearbox shows that multifunctioning can lead to extreme levels of compactness. It also shows that innovative ideas can create multifunctioning.

## 4. Human Foot Joints

### 4.1. Foot Anatomy

**Figure 6:**
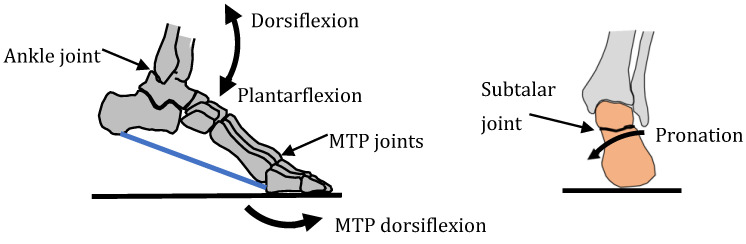
Main joints in the foot.

The foot contains approximately 26 bones, 33 joints and over 100 tendons, muscles and ligaments [57]. The main joints used in locomotion are the ankle joint, subtalar joint and MTP joint (Figure 6). The ankle joint is used to produce plantarflexion and dorsiflexion during walking and running. The subtalar joint enables pronation which helps with reducing shock absorption [58]. The MTP joints enables the foot to stand on the toes which is required for pushing off in walking and running.

**Figure 7:**
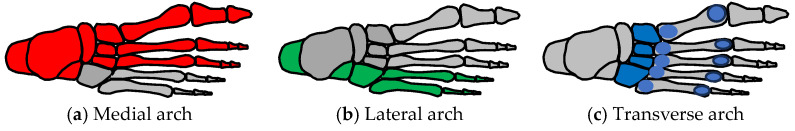
Arches of the foot.

There are three arches in the foot [57], as shown in Figure 7. The medial arch goes through the three biggest toes to the heel bone (Figure 7a). The lateral arch goes through the two smallest bones to the heel bone (Figure 7b). The transverse arch goes across the foot at the midfoot and also at the MTP joints (Figure 7c). The arches give three-point contact with the ground when standing. The three points of contact are at the bottom of the heel, the base of the big toe and the base of the little toe (Table 3).

### 4.2. Requirements and Objectives

Table 3 summarises the four major functions of the foot and the aspects of the sub-system design that make multifunctioning possible. The design objective of the foot is to have a compact design that can perform the four functions shown in Table 3. This objective is challenging because stiffness and flexibility are contradictory requirements.

### 4.3. Foot Functions and Common Parts

The foot needs to form a stiff lever during push-off to transmit the large forces required for walking and running [57]. In one study, the force in the Achilles tendon was found to be over eight times the body weight for jumping [60]. During push-off the medial arch is very stiff with a stiff big toe which points forwards. When landing, the foot transforms into a flexible lever in order to avoid large shock loads [61]. The subtalar joint is a key mechanism for reducing loads by producing a pronation movement which increases the time of landing. Loads are also reduced through the flexibility of the ligaments and tendons in all three of the arches. For example, the spring ligament in the medial arch is capable of absorbing significant energy during landing [62]. 

A third function of the feet is to be able to stand on the ball of the feet by dorsiflexion of the MTP (metatarsophalangeal) toe joints (Figure 6). Whereas running involves pressing on the base of the big toe, standing on the ball of the feet involves pressing on two points—the base of the little toe as well as the base of the big toe. Standing on the ball of the feet is important not just for reaching up high but also for changing direction during locomotion. The fourth function of three-point contact creates a clear interface with the ground during standing and aids balance. For example, it makes it possible to stand on one leg by placing the centre of gravity of the body through the triangular footprint. The three arches of the foot give three points of contact which are (i) the bottom of the heel; (ii) the base of the big toe and (iii) the base of the little toe. The triangle footprint is maximised by maximising by the distance between the three points of contact. The medial arch plays an important role in all four functions.

### 4.4. Actuation and Fine-Tuning

The bottom of the foot senses local pressures during locomotion [63] so that the nervous system can quickly make corrective adjustments to leg and foot positions to maintain stability. The medial arch is also closely aligned to the tibia bone via the talus bone so that the power of the leg muscles is focused on the medial arch. It can be seen in Table 3 how the talus aligns exactly with the medial arch. The transverse arch also plays a significant role in producing a stiff lever [64].

### 4.5. Integration, Reconfiguration and Miniaturisation

The bones, ligaments, muscles and nerve pathways of the three arches are highly integrated. A clear example of reconfiguration occurs when the foot changes from a stiff lever to a flexible lever by changes in muscle tension and changes in ligament tension [65]. Like the wrist joint there is miniaturisation in the sensors, nerves, blood vessels and lubrication system. The layout of the foot joints represents a unique solution that performs multiple functions with multiple aspects of fine-tuning and integration. 

### 4.6. Multifunctioning Robotic Feet

**Figure 8:**
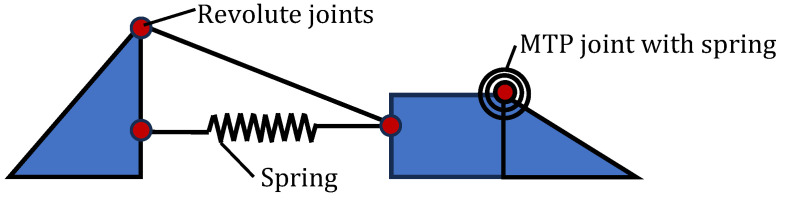
Bioinspired foot with flexible arch and MTP joint [68].

**Figure 9:**
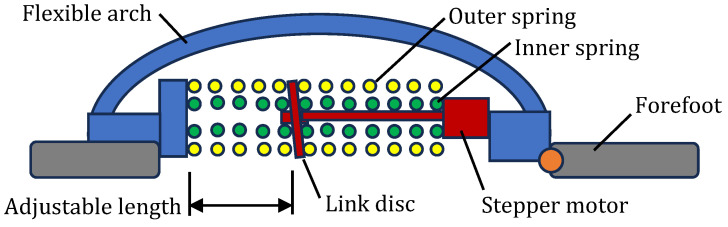
Bioinspired foot with tuneable arch stiffness [69].

**Figure 10:**
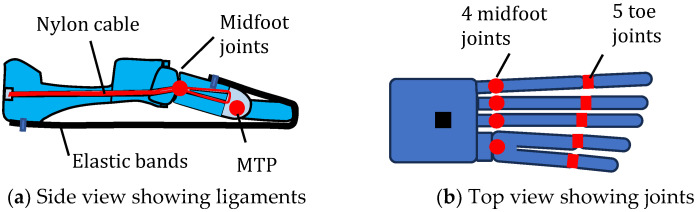
Bioinspired foot with midfoot and MTP joints [70].

Producing a robotic foot that mimics the human foot is very challenging because of the high complexity involved. Whereas hip and knee joints are commonly replaced with engineered joints, joints of the foot such as the ankle joint and MTP joints are not commonly replaced [66]. However, several researchers have recommended that the design of the human foot be copied to produce better prosthetic joints [67]. There have been several bioinspired designs with recent examples being [68,69,70,71]. Figure 8 shows a design with a flexible arch and a spring-loaded MTP joint [68]. Figure 9 shows a design with a tuneable stiffness feature which attempts to mimic the fine-tuning of stiffness in the human foot during the gait cycle [69]. This design has two concentric springs connected by an adapter which can be moved lengthwise by a stepper motor. The longitudinal position of the adapter changes the number of active coils in the outer spring. Figure 10 shows a robot foot concept where the five individual MTP joints are copied to enable close conformity of the foot with the ground [70].

Table 4 summarises the functionality of the three robotic foot concepts. Whilst all functions are covered across the three designs, no design performs all the functions.

## 5. Skeletal Muscle

### 5.1. Muscle Anatomy

Skeletal muscle is arranged in parallel bundles of myofibrils that contain many contractile units which give the tissue a striped appearance. Skeletal muscle is usually anchored by tendons to bones and is used to make skeletal movements for posture and locomotion. In skeletal muscle, contraction is stimulated by electrical impulses transmitted by the motor nerves.

### 5.2. Requirements and Objectives

Table 5 summarises the four major functions of muscle and the aspects of system design that make multifunctioning possible. Muscle–tendon units have been long recognised as a remarkable multifunctioning tissue [5]. The main design objective is to provide the right level of actuation (force and extension) for each joint whilst simultaneously providing the ideal shape for the animal. Additional functions include providing protective barriers in the right places and allowing for efficient heat transfer with blood vessels. As well as these major functions, there are other minor functions such as forming an elastic energy storage system through tendons [72] and providing a place for position sensors [73].

### 5.3. Actuator Functions and Common Parts

The primary function of muscle is actuation. Muscle is well known to be an efficient high strain actuator with high power density [74]. Animal skeletal muscle comes in two main types: red slow twitch and white fast twitch. Red muscle produces forces required for slower and sustainable movements whilst white muscle produces the forces required for rapid movements, such as sprints. Many fish species also have an intermediate pink muscle which is between the two types of muscle.

**Figure 11:**
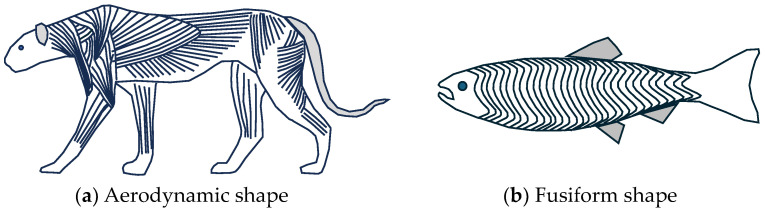
The shape of animal bodies is mainly defined by muscles and tendons.

Muscle also plays a crucial role in providing shape to the body of an animal. This is often an unappreciated function. In the case of land animals, the muscles form a shape that is ideal for locomotion such as an aerodynamic shape for fast animals (Figure 11a). In the case of fish, the muscles often form a precise hydrodynamic fusiform shape (Figure 11b). These shape objectives are met at the same time as creating a muscle mass that is ideal for actuating joints. Skeletal muscle is the main constituent of most animal bodies. Muscle accounts for up to 70% of the mass of fish [75] and 45–55% of the mass of most land mammals [76]. In the case of healthy adult humans, skeletal muscle accounts for 33–43% of body mass [77]. Muscles are also located so as to minimize the moment of inertia of limbs, especially legs. Tendons also play an important role in placing muscle away from joints, such as with the Achilles tendon.

Cushioning and providing a protective barrier are also important functions. For example, the muscles for the knee joint provide a thick cushion around the complete circumference of the femur bone (Table 5). When an animal falls to the ground, the muscles give a degree of protection to bones and organs. For example, when a human falls, they instinctively put their arms out and land on the thick thenar and hypothenar mounds on the palms of the hand. The thenar mound is formed at the base of the thumb by the intrinsic muscles of the thumb. The hypothenar mound is formed by muscles associated with the little finger. These muscle mounds act as shock absorbers. Even though muscles bruise, this is an injury that can be fully recovered from. 

Skeletal muscle also plays a major role in homeostasis by heating the blood [78]. As muscle contracts, there are chemical reactions that generate heat which is transferred to the blood. The tendons function as an energy storage system for fast movements or oscillating movements such as flapping [79]. During fast movements, tendons buffer the work done on muscle by temporarily storing elastic energy then releasing this energy to assist the movement. The tendon deforms in a linear fashion due to the inter-molecular sliding of collagen triple helices.

### 5.4. Fine-Tuning

The nervous system has fine control over muscle force levels and force take-up because a muscle bundle is typically broken down into dozens of muscle units (MU). Muscle units are usually recruited by the nervous system from the smallest to largest which results in a smooth increase in force [80]. Another aspect of fine-tuning is the way that each muscle size is simultaneously optimal for actuation and shape.

### 5.5. Integration, Reconfiguration and Miniaturisation

Skeletal muscle is remarkably integrated with each muscle containing dozens of muscle units, each one with individually integrated nerve pathways for control and sensing. In addition, there is integration of the blood vessels. The shivering response to cold is an example of multifunctioning through reconfiguration. When a person gets cold, the body will go into shiver mode, causing muscles to contract and thus creating heat that will be transferred to the blood [81].

### 5.6. Bioinspired Muscle

There has been significant progress in the development of bioinspired muscle-like actuators [82,83,84,85] which are sometimes close to matching the performance of animal muscle in terms of the power density. However, bioinspired muscles are not yet multifunctional in providing shape or a protective barrier [74]. Future developments should investigate developing bioinspired muscle with these multifunctioning properties.

**Figure 12:**
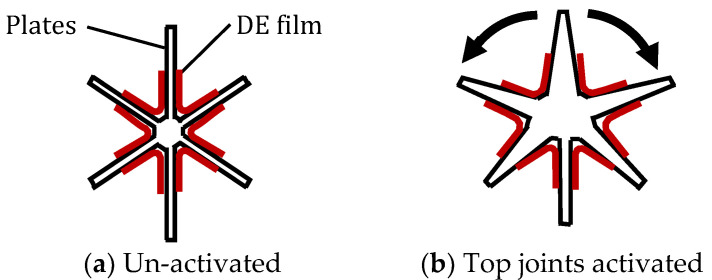
Bioinspired bilateral muscle in star configuration [86].

A recent development in bioinspired muscle actuators is that of bilateral dielectric elastomer (DE) actuators assembled in back-to-back configurations, as shown in Figure 12 [86]. This concept has been inspired by the antagonistic pairs of muscles used in animal joints. One configuration involves a star-shaped wheel where each pair of plates can be individually opened out to create asymmetry in the wheel (Figure 12b). When a voltage is applied to the electrodes on both sides of a pair of plates, the DE film is subjected to Maxwell stress and shrinks in the direction of the electric field and expands in the direction perpendicular to the electric field. The star wheel can be used to create motion of a robotic vehicle by sequentially moving each pair of plates in turn [86].

## 6. Discussion

### 6.1. The Importance of Multifunctioning in Engineering Systems

There is growing recognition that multifunctioning can play an important role in the optimisation of human-engineered systems. Multifunctioning has been identified as particularly desirable in aerospace systems where there are tight limits on the mass and size of sub-systems [87]. Recently a multifunctioning morphing wing has been developed that creates optimal lift-to-drag ratios for different speeds [88]. Miniaturisation is seen as a crucial goal for many medical devices [89,90,91] and multifunctioning is a key means for achieving this goal. Multifunctioning is now seen as an important means of achieving miniaturisation in electronics [92].

The benefits of multifunctioning can be seen in the development of mobile phones which have transitioned from bulky assemblies to compact designs with far more functionality. Modern smart phones pack a large amount of functionality in a small volume, partly by miniaturisation, but also by using multifunctioning materials and sub-systems. Multifunctioning is found in screens that are touch sensitive as well as providing a visual display and protective shield. Sensors are often multifunctioning, such as magnetometers that act as metal detectors as well as a compass. Smart phone processors are multitasking with multiple cores. One of the lessons of mobile phones is that multifunctioning and miniaturisation come at a stage of mature product development and after significant financial investment. At present, the field of robotics has not reached that stage of maturity.

### 6.2. Energy Benefits of Multifunctioning Limb Joints

Reductions in joint mass not only reduces the overall mass of a robot but also the moment of inertia of the limbs. This has a significant effect on the energy demands of locomotion. Since the legs move backwards and forwards during locomotion, minimising the moment of inertia of the legs helps minimise energy demands. The torque, *T*, required to cause angular acceleration of the legs is proportional to the moment of inertia (*T* = *Iα*). The moment of inertia of the leg, *I*, is most influenced by masses, *m_n_*, furthest from the hip joint because the radius of gyration term, *k_n_*, is squared in the equation for *I* (*I* = Σ*m_n_k_n_*^2^). This means that the masses of the joints in the foot have a large effect on the torque required. In one study it was found that a third of the energy required for walking goes into moving the legs backwards and forwards [93].

The proportion of energy required to swing the legs is higher for faster walking and running speeds because the torque, *T*, required to swing the legs is also proportional to the angular acceleration, α. In one experimental study it was found that adding just 100 g to the mass of each running shoe increased the cost of transport (kJkg^−1^km^−1^) by up to 7.4% for high intensity running at 85% of the second ventilatory threshold [94]. The extra 200 g represented a small increase in the mass of the adult of around 0.3%. If a mass of 200 g was added to the torso it would be expected that the increase in energy for locomotion would be only 0.3% since the energy required for running is approximately proportional to the body mass [95]. This confirms that the leg inertia has a large influence on the energy demands of locomotion, especially at high speeds.

As well as reductions in the moment of inertia, a compact joint also results in a low frontal area and therefore lower drag. Therefore, there are very large energy benefits to be gained from minimising the mass and volume of limb joints through multifunctioning.

### 6.3. Comparison between Animal and Robot Joints

As shown in Section 2, Section 3 and Section 4, all three joints considered—wrist, knee and foot joints—combine extreme compactness with high functionality. This helps to explain the highly impressive agility of animals. None of the joints have a fully bioinspired engineering counterpart. However, there are some aspects of the joints that have been copied, as illustrated in the relevant sections. The knee joint has far fewer bones than the feet and hands (3 compared to 26/27) which makes bioinspired design more feasible. However, the knee joint has a very complex geometry and the meniscus itself is a very complex component. Much of the functionality and design of the knee joint has been copied with the exception of the rotation function around the tibia axis. If future robots are required to move with agility such as running on uneven ground, then copying the rotation function will be required. Wrist joints and foot joints are a long way off from being fully copied. Bioinspired feet are particularly challenging because of the need to tune stiffness during the gait cycle. A recent study has shown the vertebrate limb pattern to be highly optimal, with a key reason being the multifunctioning capability of the joints [96].

### 6.4. Strategies for Producing Multifunctioning

Biological joints reveal detailed strategies that can be employed to create multifunctioning systems. These can be considered for bioinspired systems. Some of these strategies are detailed here. 

*(i) Innovative and finely tuned designs*. Multifunctioning in joints requires innovative solutions in order to create multiple degree-of-freedom joints. Therefore, it can be expected that significant innovation is required to produce bioinspired multifunctioning joints. The requirements of multifunctioning are so exacting that fine-tuning of design is also generally required, such as the common centre of rotation in the wrist (Section 2), the geometry of the cruciate ligament 4-bar linkage in the knee (Section 3) and the precise alignment of the medial arch with the talus bone in the foot (Section 4). Therefore, it can be expected that fine-tuning is required for robotic multifunctioning joints. Indeed, this was the case for the three bioinspired designs presented in this paper for the wrist, knee and foot.

*(ii) Under-constrained and over-actuated joints*. All three joints considered have a degree of under-constraint. For example, the knee joint is free to rotate during most of the flexion range and the wrist joint is free to abduct–adduct throughout most of the flexion range. This freedom means that fine control of actuators is required for stability. The advantage of under-constraining is that it creates more freedom of movement. All three case studies considered also have joints that are over-actuated with more actuators than degrees of freedom. The advantage of over-actuating is that it creates a greater range of possible movements. All three of the bioinspired examples have a degree of under-constraining.

*(iii) Multifunctioning actuators*. All three joints benefit from the multifunctioning capabilities of animal muscle. For example, the muscles for all three joints provide a protective barrier around the bones of the joints. Despite much progress in the development of soft actuators, they are usually not optimised for additional functions such as shape and protective barriers [97].

*(iv) Reconfigurable designs.* Reconfiguration can be an effective means for multifunctioning by actively changing the properties of a sub-system such as stiffness or shape. This is particularly seen in the human foot which becomes stiff for push-off by active stiffening by the muscles and by tensioning of the plantar ligament (Section 4). Reconfiguration is used in electronics [98] and active suspension systems [99] but is not commonly used in robotic joints. So, there is an opportunity for using this strategy. 

*(v) Segmented joints with high DoF.* The feet and hands of vertebrates have segmented layouts with multiple bones and many joints. This layout allows for complex geometries and movements with high degrees of freedom. Copying such designs is very challenging because of the difficulty in copying ligament-type connections. However, the benefits of achieving are so high that it is worth the effort in attempting such assemblies. There has been a recent bioinspired robotic foot concept that does copy some of the individual bones of the midfoot [70]

### 6.5. Trade-Offs for Multifunctioning

Multifunctioning inevitably involves design trade-offs [100] and it is not possible to achieve the maximum performance for every function. For example, in the case of the wrist joint it is not possible to simultaneously have maximum flexion and abduction [37], and the knee joint cannot have maximum rotation and flexion at the same time [47]. The freedom of the knee joint means it can be moved in many directions. However, if the muscles get out of balance the knee can become unstable [101]. Therefore, there is a compromise between stability and functionality [101].

**Figure 13:**
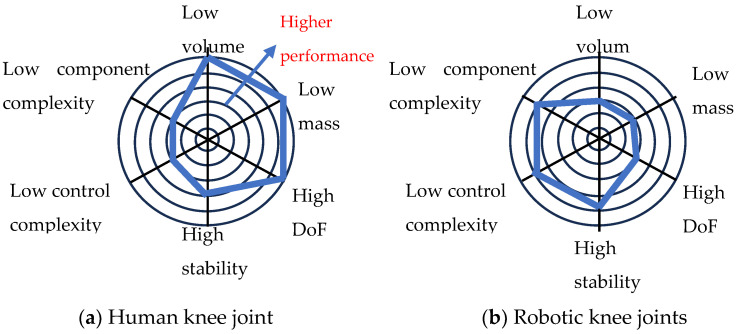
Illustrative radar trade-off charts for knee joints.

Radar trade-off charts can be used to visualise the trade-offs of functions [102]. To illustrate the trade-offs involved in multifunctioning, Figure 13a presents an illustrative trade-off chart for six aspects of performance for the human knee joint. In terms of compactness, mass and DoF, the knee performs very highly. However, this comes at the cost of complexity of parts, complexity of control and lower stability. The knee joint has a highly complex geometry in the bones and meniscus and also has complex control requiring extensive feedback of muscle positions. The under-constrained nature of the knee is a mixed blessing. On the one hand, it enables complex movements but, on the other hand, it can lead to instability. In terms of overall performance, multifunctioning is the right solution for the knee joint, but the design illustrates the trade-offs involved in multifunctioning.

The equivalent trade-off chart for robotic knee joints is shown in Figure 13b. In contrast to animals, robotic knee joints perform less well in terms of compactness, mass and DoF but better in terms of complexity and stability [2]. For robots to achieve high levels of compactness and light weight, it may be inevitable that there will be some compromises in terms of complexity and/or stability.

### 6.6. Design Process for Multifunctioning

Traditional design methods generally do not encourage multifunctioning design. For example, morphological charts imply that all the individual subfunctions should have their own individual function carrier because separate boxes are allocated for each sub-function [103,104,105]. Whilst it is normal for each component to have ‘secondary’ functions such as having a protective coating, it is assumed they have one primary function. There have been recent attempts to develop design methods specifically for achieving multifunctioning systems [106,107,108]. These methods encourage the designer to investigate the possibility of multifunctioning solutions at the conceptual design stage through abstract visualization of function carriers that are multifunctioning.

### 6.7. The Emergence of Multifunctioning in Biology

Complexity in biological systems is sometimes labelled as an emerging property [109]. However, it is very difficult to explain how a multifunctioning system could emerge from an initially single-functioning system because the first single-functioning system would have to be one of the very few solutions that could lead to a later multifunctioning system [106]. When discussing the origin of mechanical linkage mechanisms in animal joints, Muller has stated that it is very difficult to see how complex mechanical linkage systems can be developed in a bottom-up step-by-step process [53]. Therefore, multifunctioning in biological systems such as limb joints presents a major challenge of irreducible complexity for evolutionary biologists [110].

## 7. Conclusions

The joints of the wrist, knee and foot contain remarkably sophisticated multifunctioning designs that outperform the best robotic devices. Multifunctioning in robotic joints can produce very important benefits of compactness, low mass, low moment of inertia and low drag. A key finding of this study is that multifunctioning in animal joints is achieved through various means: a high number of degrees-of-freedom, under-constrained assemblies, over-actuation, multifunctioning parts, miniaturisation and reconfiguration. Muscle is a remarkable actuator which performs other important functions including providing shape and protection and a heat source. Whilst there has been some progress in creating multifunctioning robotic joints, the progress is limited and there is much potential for bioinspiration.

## Figures and Tables

**Table 1:** Summary of wrist functions and how they are achieved.

Main Functions	Flexion–Extension	Abduction–Adduction	Longitudinal Load Paths	Carpal Arch
Schematic	Midcarpal joint Radiocarpal joint 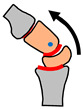	Midcarpal joint Radiocarpal joint 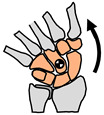	Two load columns Two arches 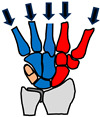	Arch in cross-section of hand 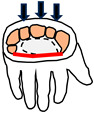
Common parts (wrist bones)	All of top row Three of bottom row 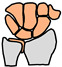	Three of top row Three of bottom row 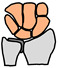	All of top row Two of bottom row 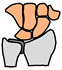	All of top row Two of bottom row 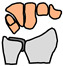
Actuation [41]	Multifunctioning muscles	Multifunctioning muscles		
Fine tuning	Common centre of rotation 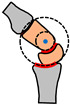	Common centre of rotation 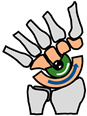	Alignment of metacarpals with top row of wrist bones.	Arch segments aligning simultaneously in two different planes
Integration	Network of 8 bones and >25 ligaments	Multiple joint interfaces (Capitate = 5)	Capitate styloid process	Carpal arch integrated with tendons/nerves
Reconfiguration			Muscle tension to stiffen wrist during loading	Muscle tension to stiffen wrist during loading
Miniaturisation	Sensors, nerves, blood vessels, lubrication	Sensors, nerves, blood vessels, lubrication	Sensors, nerves, blood vessels, lubrication	Sensors, nerves, blood vessels, lubrication

**Table 3:** Summary of foot functions and how they are achieved.

Main Functions	Stiff Lever (for Push-Off)	Flexible Lever (for Landing)	Stand on Ball of Feet	Three-Point Contact (for Standing)
Schematic	Medial arch 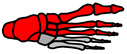	All arches Pronation 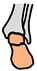	Five MTP joints 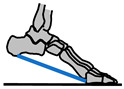	All arches 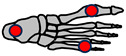
Common parts	Medial arch	Medial arch Lateral arch Transverse arch	Medial arch Lateral arch Transverse arch	Medial arch Lateral arch Transverse arch
Actuation [59]	Seven muscles over-actuated	Four muscles over-actuated	Twenty-two muscles over-actuated	n/a
Fine tuning	Alignment of talus bone with medial arch	Spring ligament alignment	Alignment of five MTP joints	Maximised spacing of contact points
Integration	Bones of medial arch	Integration of three arches	Integration of three arches	Integration pf three arches
Reconfiguration	Tightening of muscles and ligaments			
Miniaturisation	Sensors, nerves, blood vessels, lubrication	Sensors, nerves, blood vessels, lubrication	Sensors, nerves, blood vessels, lubrication	Sensors, nerves, blood vessels, lubrication

**Table 4:** Functionality of three bioinspired robotic feet.

Foot Example	Schematic	Flexible Arch	Tuneable Stiffness	MTP Joint	Individual Toes
[68]	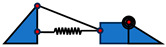	Y	N	Y	N
[69]	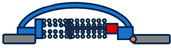	Y	Y	Y	N
[70]	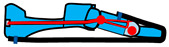	Y	N	Y	Y

**Table 5:** Summary of muscle functions and how these are achieved.

Main Functions	Actuator (e.g., Move Joints)	Shape (e.g., Optimise Hydrodynamics)	Cushioning (e.g., Protect Organs & Bones)	Heat Source (for Blood Vessels)
Schematic	Muscle contraction 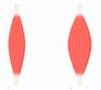	Muscle defines animal shape 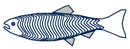	Protective barrier Thigh section 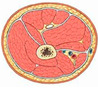	Heat transfer 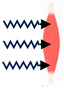
Common parts	Muscle and tendon	Muscle and tendon	Muscle and tendon	Muscle
Fine-tuning	MUs recruited from smallest to largest three types of muscle	Animals: aerodynamic shape Fish: hydrodynamic shape	Distribution Self-healing	Maximal heat transfer
Integration	Nerves for each muscle unit	Muscles with bones and organs	Muscles with bones and organs	Blood vessels
Reconfiguration				Shivering for generating heat [9]
Miniaturisation	Sensors, nerves, blood vessels, lubrication	Sensors, nerves, blood vessels, lubrication	Sensors, nerves, blood vessels, lubrication	Sensors, nerves, blood vessels, lubrication

## Data Availability

Data for this review paper can be found within the paper and the references.
